# Conversion of monoculture cropland and open grassland to agroforestry alters the abundance of soil bacteria, fungi and soil-N-cycling genes

**DOI:** 10.1371/journal.pone.0218779

**Published:** 2019-06-27

**Authors:** Lukas Beule, Marife D. Corre, Marcus Schmidt, Leonie Göbel, Edzo Veldkamp, Petr Karlovsky

**Affiliations:** 1 Molecular Phytopathology and Mycotoxin Research, Faculty of Agricultural Sciences, University of Goettingen, Goettingen, Germany; 2 Soil Science of Tropical and Subtropical Ecosystems, Faculty of Forest Sciences and Forest Ecology, University of Goettingen, Goettingen, Germany; Consiglio per la Ricerca e la Sperimentazione in Agricoltura, ITALY

## Abstract

Integration of trees in agroforestry systems can increase the system sustainability compared to monocultures. The resulting increase in system complexity is likely to affect soil-N cycling by altering soil microbial community structure and functions. Our study aimed to assess the abundance of genes encoding enzymes involved in soil-N cycling in paired monoculture and agroforestry cropland in a Phaeozem soil, and paired open grassland and agroforestry grassland in Histosol and Anthrosol soils. The soil fungi-to-bacteria ratio was greater in the tree row than in the crop or grass rows of the monoculture cropland and open grassland in all soil types, possibly due to increased input of tree residues and the absence of tillage in the Phaeozem (cropland) soil. In the Phaeozem (cropland) soil, gene abundances of *amoA* indicated a niche differentiation between archaeal and bacterial ammonia oxidizers that distinctly separated the influence of the tree row from the crop row and monoculture system. Abundances of nitrate (*napA* and *narG*), nitrite (*nirK* and *nirS*) and nitrous oxide reductase genes (*nosZ* clade I) were largely influenced by soil type rather than management system. The soil types’ effects were associated with their differences in soil organic C, total N and pH. Our findings show that in temperate regions, conversion of monoculture cropland and open grassland to agroforestry systems can alter the abundance of soil bacteria and fungi and soil-N-cycling genes, particularly genes involved in ammonium oxidation.

## Introduction

Modern alley cropping systems are innovative agroforestry systems, where rows of fast growing trees are planted alternately with rows of annual crops [1]. Spatial arrangement of the tree and crop components allows ecological interactions between them, which can increase the overall efficiency of the resource use if the trees and crops are not competing for the same resources [2,3]. For example, due to deeper root growth, most tree species can access water and nutrients from deeper soil layers than the associated crops [3,4]. Deep-rooting trees are thus capable of providing a ‘safety-net’ for leached nutrients from the crop rhizosphere by deep-root nutrient uptake [5–8].

In contrast to monoculture agriculture, agroforestry can be more sustainable by conserving and improving soil quality through increasing soil organic C and nutrient availability [9,10] and its potential for C sequestration [10]. Increases in organic C stocks are mainly due to litter input and root decay of the tree rows [11]. Inclusion of N_2_-fixing trees further enhances physico-chemical as well as biological properties of soil and thus contributes to an improvement of soil fertility [12–14]. Most trees in temperate alley cropping systems are fast-growing trees like *Salix* and *Populus* species, suitable for biomass production.

Soil microbial community structure and function are shaped by resource availability, which in turn is controlled, among other factors, by the quantity and quality of plant litter input as well as root exudation and decay [15,16]. Thus, it is likely that trees integrated in agroforestry systems affect soil microbial communities on both structural and functional levels. For example, a recent study in three poplar-based temperate agroforestry systems found greater soil fungal C-to-bacterial C ratios in the tree rows than the crop rows [17], probably due to increased input of lignin- and suberin-rich tree residues. Soil enzymatic activities and substrate utilization patterns of soil microbial communities also indicate that microbial communities in the tree and crop rows of agroforestry systems are functionally different [11,18,19]. Furthermore, other studies have reported beneficial effects of agroforestry on the abundance and diversity of the soil microbial community [12,20,21]. A recent study investigated for the first time the soil bacterial community structure in temperate agroforestry systems using next generation sequencing [22]. They found that the integration of trees increases the abundance and species richness of soil bacteria, which can have several causes. Plant diversity and productivity affect soil microbial processes such as N mineralization [23] and the nitrification-denitrification pathway [24]. For example, increased crop diversity by intercropping of maize (*Zea mays*) with peanut (*Arachis hypogaea*) resulted in an increase in *nifH*, ammonia-oxidizing archaea (AOA) and ammonia-oxidizing bacteria (AOB) *amoA* and *narG* abundance, whereas *nirK*, *nirS* and *nosZ* clade I remained unaffected [25]. Although soil microbial responses to agricultural management are highly complex [26] and not uniform among microbial groups [27], there is substantial evidence that agricultural intensification is one of the main drivers of observed losses in soil microbial diversity and connectivity [28–30]. The decreased complexity of intensive agricultural systems contributes to the loss of soil microbial biodiversity and is expected to negatively affect soil processes like nutrient cycling and retention [31,32]. The increase in system complexity associated with agroforestry is likely to alter soil microbial community structure and function, and thus soil-N-cycling.

Several studies have shown that temperate agroforestry systems affect N cycling and enzymatic activities in soils; however, associated changes in the abundance of microbial communities involved in soil-N cycling as well as soil fungi and bacteria remain to be elucidated. The main aim of this study was to test whether the abundance of genes encoding enzymes involved in soil-N cycling differ under temperate monoculture and agroforestry cropland and open grassland and agroforestry grassland. A further aim was to assess differences in the abundance of bacterial and fungal populations by quantifying bacterial 16S rRNA and fungal 18S rRNA genes. We hypothesized that the tree row in the agroforestry systems increases the above- and belowground litter input and nutrient cycling, which in turn i) changes the abundance of functional genes involved in nitrification and denitrification, and ii) increases relative fungal abundance. In contrast to prior studies analyzing substrate utilization patterns and enzymatic activities of soils under laboratory conditions, this is, to our knowledge, the first field-based study investigating differences in the abundance of genes involved in soil-N cycling between temperate monoculture and agroforestry cropland and open grassland and agroforestry grassland.

## Materials and methods

### Study sites

We selected two sites at which monoculture cropland or open grassland was converted into agroforestry systems with an alley cropping design. The cropland site with a Phaeozem soil was located near Dornburg, Thuringia, Germany (51° 00’ 40” N, 11° 38’ 46” E, 289 m asl) (S1A Fig). The site has a mean annual precipitation of 608 ± 21 mm and mean annual air temperature of 9.9 ± 0.1°C (mean ± SE during 1981–2010; climate station at Jena (station ID: 2444) of the German Meteorological Service). During our sampling year (2017), the site received 648 mm precipitation and mean temperature was 10.9°C. In 2007, a monoculture cropland was converted into an agroforestry system by planting 12-m wide poplar rows (clone Max1; *Populus nigra* x *P*. *maximowiczii*), alternated with 48-m wide crop rows, forming an alley cropping pattern (S1C Fig). The tree rows we established from poplar cuttings, planted by hand using a dibble bar. In January 2015, the aboveground biomass of trees in the agroforestry system was harvested for the first time since establishment. An adjacent monoculture cropland was not converted and used as a reference with identical crops, management and fertilization rates. Both monoculture and agroforestry had the following crop rotation in the four years previous to our sampling: winter wheat (*Triticum aestivum*) (2014) / summer barley (*Hordeum vulgare*) (2015) / summer barley (2016) / oilseed rape (*Brassica napus*) (2017). On 24 August 2016, both the agroforestry crop rows and the reference monoculture cropland were fertilized with cattle manure (30 m^3^ ha^-1^, equivalent to 105 kg N ha^-1^–19.5 kg P ha^-1^–97.2 kg K ha^-1^). In accordance with common temperate agroforestry practice, the tree rows were not fertilized [33]; however, due to the close spatial proximity of the unfertilized tree rows to the fertilized crop rows, the trees may have taken up fertilizer.

The grassland site with Histosol and Anthrosol soils is located near Mariensee, Lower Saxony, Germany (52° 33’ 49” N, 9° 28’ 9” E, 42 m asl) (S1A Fig). This site has a mean annual precipitation of 661 ± 20 mm and mean annual air temperature of 9.6 ± 0.2°C (1981–2010; climate station at Hannover (station ID: 2014) of the German Meteorological Service). In 2017, annual precipitation was 822 mm and annual mean temperature was 10.4°C. At this site, part of a continuous open grassland (at least 30 years old) was converted into an agroforestry system in 2008, by planting 11-m wide rows of willow (clone Tora; *Salix schwerinii* x *S*. *Viminalis*), alternated with 48-m wide grass rows, also in an alley cropping pattern (S1B Fig). Willow cuttings were planted by hand using a dibble bar to protect the grass sward. In January 2016, the aboveground biomass of trees in the agroforestry system was harvested for the first time since planting. The grassland in both unconverted open grassland and the agroforestry system were managed in the same way: grasses were cut once a year and the last fertilizer application (15 m^3^ ha^-1^ biogas plant residue containing 50 kg N ha^-1^) before sampling was on 10 November 2015. As was the case in the cropland agroforestry system, the tree rows were not fertilized [33], but the trees may have taken up applied fertilizer due to their close spatial proximity to the fertilized grass rows. The composition of the grass sward was the same in the two systems, consisting of *Lolium perenne*, *Festuca pratensis*, *Phleum pratense*, and *Poa pratensis*.

### Experimental design and soil sampling

At the cropland site, we established four replicate plots in both the agroforestry and monoculture. Each replicate plot of the agroforestry system included soil sampling locations in the tree row and the crop row at distances of 1 m, 4 m and 7 m from the tree row (S1A Fig). In the monoculture system, soil samples were taken in the middle of each replicate plot. At the grassland site, we established in each of the two soil types three replicate plots in agroforestry and three in open grassland. At this site, soil sampling locations also included the tree row and the grass row at distances of 1, 4 and 7 m from the tree row, whereas in the open grassland soil samples were taken in the middle of each replicate plot (S1A Fig).

We took soil samples in the top 0.05-m depth on 3 May 2017 at the cropland site (Phaeozem soil) (252 days after fertilisation) and on 11 April 2017 at the grassland site (Histosol and Anthrosol soils) (518 days after fertilization). Three subsamples were taken per sampling location of each replicate plot. Soils were sampled using sterile 15-mL polypropylene Falcon tubes (Sarstedt Ag & Co, Nümbrecht, Germany) without any hand contact with the soil. The soil samples were frozen (-20°C) in the field and immediately stored at -20°C upon arrival at the laboratory. Starting the following day, the soil samples were freeze-dried for 72 h and vortexed in the same Falcon tubes with two tungsten carbide beads of 6 mm diameter for 60 s using a vortexer (HS120209, Heathrow Scientific, Vernon Hills, USA) at maximum power. Finely ground subsamples were pooled to obtain one mixed soil sample per sampling location of each replicate plot. Pooled soil samples were stored at -20°C until DNA extraction.

### DNA extraction from soil

DNA was extracted according to Beule *et al*. [34] from 30 mg soil. Briefly, finely grounded soil was suspended in 1 ml of CTAB buffer with proteinase K and incubated at 42°C and subsequently at 65°C for 10min each. Two chloroform-isoamylalcohol extractions with an intermediate phenol extraction were conducted, and DNA was precipitated with polyethylene glycol and pelleted by centrifugation. DNA pellets were washed twice with ethanol, re-suspended in 50 μl of TE buffer (10 mM Tris, 1 mM EDTA, pH 8.0), and incubated at 42°C for 2 h to facilitate dissolution of DNA. Concentration and quality of the DNA were assessed by agarose gel electrophoresis using 1.7% (w/v) agarose gels, and DNA extracts were stored at -20°C until analysis.

### Real-time PCR

Different primers were used for real-time PCR (qPCR) to amplify parts of the bacterial 16S rRNA and fungal 18S rRNA gene as well as microbial genes encoding subunits of enzymes involved in nitrification: AOA *amoA*, AOB *amoA and nxrB* (Table 1; for the corresponding nitrification steps see S2A Fig) and denitrification: *napA*, *narG*, *nirK*, *nirS*, *nosZ* clade I and *nosZ* clade II (Table 1; for the corresponding denitrification steps see S2B Fig).

**Table 1:** Target genes with corresponding oligonucleotide primers used for qPCR assays.

Target gene	Primer pair	Expected amplicon size (bp)	Reference
Bacterial 16S ribosomal RNA (16S rRNA)	341F	194	[35]
	534R		
Fungal 18S ribosomal RNA (18S rRNA)	FR1	350	[36]
	FF390		
Crenarchaeal ammonia monooxygenase α subunit (AOA *amoA*)	CrenamoA23f	628	[37]
	CrenamoA616r		
Bacterial ammonia monooxygenase α subunit (AOB *amoA*)	amoA-1F	491	[38]
	amoA-2R		
Nitrite oxidoreductase β subunit of *Nitrobacter*-like spp. (*nxrB*)	NxrB-1F	380	[39]
	NxrB-1R		
Proteobacterial periplasmic nitrate reductase catalytic subunit (*napA*)	V17m	152	[40]
	napA4r		
Proteobacterial membrane-bound nitrate reductase catalytic subunit (*narG*)	narG-f	173	[40]
	narG-r		
Cu-nitrite reductase catalytic subunit (*nirK*)	nirK876F	165	[41]
	nirK1040R		
*Cd*_*1*_-nitrite reductase catalytic subunit (*nirS*)	cd3aF	410	[42,43]
	R3cd		
Nitrous oxide reductase catalytic subunit (*nosZ* clade I)	nosZ2F	267	[44]
	nosZ2R		
Nitrous oxide reductase catalytic subunit (*nosZ* clade II)	nosZ-II-R	698	[45]
	nosZ-II-F		

Standard curves for qPCRs were generated in two replicates using 1:3 serial dilutions in 0.5X TE buffer. Standards of Bacterial 16S rRNA, fungal 18S rRNA, *nirK*, *nirS*, *nosZ* clade I and *nosZ* clade II genes were obtained as described previously [46]. AOA *amoA* gene was obtained from an environmental clone, cloned in plasmid pGEM-T (Promega, Mannheim, Germany) and multiplied in *Escherichia coli* JM109. AOB *amoA*, *nxrB*, *napA* and *narG* gene standards were obtained from *Nitrosomonas europeae* DSM 28437, *Nitrobacter winogradskyi* DSM 10237 and *E*. *coli* DH񐎱 (*napA* and *narG*), respectively. Genomic DNA of *E*. *coli* DH5񐎱 was extracted using a CTAB protocol [47]; *Nitrosomonas europeae* DSM 28437 was ordered as genomic DNA dissolved in TE buffer from the German Collection of Microorganisms and Cell Cultures (DSMZ). *Nitrobacter winogradskyi* DSM 10237 was grown in mixotrophic *Nitrobacter* medium (DSMZ Medium 756a) and genomic DNA was isolated from a 2-ml aliquot of the culture according to Wilson [48].

All qPCR reactions were carried out in a CFX 384 Thermocycler (Biorad, Rüdigheim, Germany) in 384-well microplates and conditions are listed in S2 Table. Amplification was performed with 1:100 dilutions of the DNA extracts in 4 μl reaction volume that contained the following: 3 μl mastermix (buffer (10 mM Tris-HCl, 50 mM KCl, 1.5 mM MgCl_2_, pH 8.3 at 25°C); varying MgCl_2_ concentrations (S2 Table); 100 μM of each deoxyribonucleoside triphosphate (Bioline, Luckenwalde, Germany); 0.3, 0.5 or 1.0 μM of each primer (S2 Table), 0.1X SYBR Green I solution (Invitrogen, Karlsruhe, Germany); 1 mg/ml bovine serum albumin; 0.03 u Hot Start *Taq* DNA Polymerase (New England Biolabs, Beverly, Massachusetts, USA)); and 1 μl template DNA solution or double-distilled H_2_O for negative controls. Amplification of each standard DNA concentration and negative control was performed in two replicates. For *napA*, *narG*, *nirK*, *nirS* and *nosZ* clade I genes, six touchdown cycles were run at the beginning, consisting of 20s denaturation at 94°C, 30s annealing beginning at 66°C, 63°C, 63°C, 58°C and 65°C for *napA*, *narG*, *nirK*, *nirS* and *nosZ* clade I genes, respectively, with a decrease of the annealing temperature by 1°C per cycle, and 30s (15s for *napA* and *narG*) extension at 68°C. To obtain melting curves, samples were heated to 95°C for 60s and cooled to 55°C for 60s followed by a temperature increase from 55°C to 95°C by 0.5°C per cycle with continuous fluorescence measurement.

### Soil properties

Physical and biochemical properties of soil were measured on soil samples taken in immediate proximity to the sampling locations for soil DNA extraction. Soil samples for the measurement of water-filled pore space (WFPS), plant-available P and total extractable N in the top 0.05-m depth were taken on same day that samples for DNA extraction were taken. WFPS was calculated from the measured gravimetric moisture content and soil bulk density, determined using the soil core method [49]. Plant-available P was the sum of resin- and bicarbonate-extractable P [50]. The concentration of P in the extracts was determined using an inductively coupled plasma-atomic emission spectrometer (ICP-AES, iCAP 6300 Duo VIEW ICP Spectrometer, Thermo Fischer Scientific GmbH, Dreieich, Germany). Total extractable N was extracted in the field by placing soil into bottles containing 150 mL 0.5 M K_2_SO_4_. Upon arrival in the laboratory, the bottles were shaken for one hour, and the extracts were filtered through pre-washed filter papers (7 μm nominal pore size) and kept frozen at -20°C until analysis. Total extractable N was measured using continuous flow injection colorimetry (SEAL Analytical AA3, SEAL Analytical GmbH, Norderstedt, Germany). Since soil pH, soil organic C, total N and exchangeable K, Mg, Mn, and Na do not vary within a few years, we report values that were measured from samples taken in October-December 2016 for the Phaeozem soil (cropland) and in August 2016 for Histosol and Anthrosol soils (grassland) for the top 0.3-m depth. Soil pH was determined from a soil:water ratio of 1:4. Soil organic C and total N were determined using a CN analyzer (Elementar Vario El, Elementar Analysis Systems GmbH, Hanau, Germany); for soil samples with pH ≥ 6.0 pre-treatment for the removal of carbonates was performed [51]. For all the exchangeable cations, soil was percolated with unbuffered 1 M NH_4_Cl and cations in the percolate were determined using ICP-AES. The values we report in S1 Table are calculated for the top 0.05-m depth.

### Statistical analysis

Each parameter was tested for equality of variance (Levene’s test) and normality of distribution (Shapiro-Wilk’s test). Differences among sampling locations within the agroforestry system (tree row, 1 m, 4 m and 7 m within the crop or grass row) and the monoculture or open grassland system for a soil type as well as among soil types for a management system were tested using one-way ANOVA with Tukey’s HSD test for parameters with normal distribution and homogeneous variance; if otherwise, we used Kruskal-Wallis test with multiple comparison extension. Statistical significance was taken at p ≤ 0.05, except for a few parameters with p > 0.05 ≤ 0.08 that are mentioned as marginally significant. We conducted a network analysis based on Spearman’s rank correlation test to assess the relationships between gene abundances and soil properties. Only correlations that were highly robust (p < 0.001) with a correlation coefficient (r) of r > |0.7| were reflected in the network analysis. This was performed using the “igraph” R-package [52]. Finally, a principle component analysis (PCA) of the gene abundances and soil properties was performed using the “factoextra” R-package [53]. All statistical analyses were conducted using R version 3.4.3.

## Field-work permission

This study was a part of the project ‘Sustainable intensification of agriculture through agroforestry’ (SIGNAL) funded by the German Federal Ministry of Education and Research (BMBF). The fieldwork included soil sampling and was carried out on private land. The local land owners of the study sites gave permission to conduct the fieldwork on their sites. Besides this, no specific permissions were required to perform the fieldwork since the study sites were not located in a protected area. This field study did not involve endangered or protected species.

## Results

### Abundance of bacterial 16S rRNA, fungal 18S rRNA and soil-N-cycling genes

**Fig 1:**
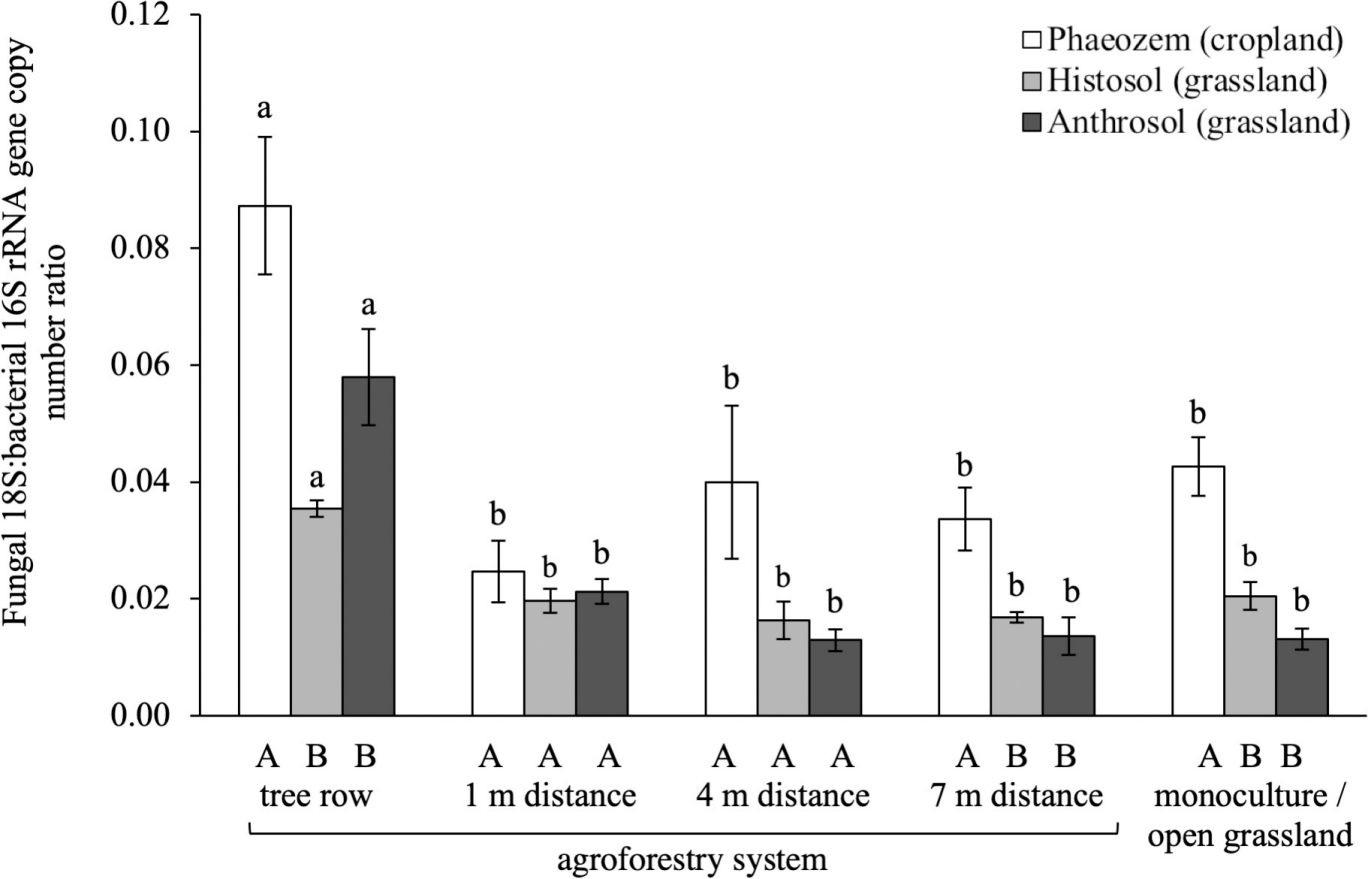
Ratio of fungal 18S-to-bacterial 16S rRNA gene abundances in soils of paired temperate monoculture and agroforestry cropland, and paired temperate open grassland and agroforestry grassland. Whiskers represent the SE (n *=* 4 for Phaeozem soil, n *=* 3 for Histosol and Anthrosol soils). Within the same soil type, means with different lowercase letters indicate significant differences among the tree row, 1 m, 4 m and 7 m within the grass or crop row of the agroforestry and the monoculture or open grassland system. Different uppercase letters indicate significant differences among soil types within the same sampling location of a management system (one-way ANOVA with Tukey’s HSD test or Kruskal-Wallis test with multiple comparison extension at p ≤ 0.05).

Bacterial 16S rRNA genes were the most abundant across the two management systems of both cropland and grassland soils, ranging from log_10_ 9.3 to 10.3 copies g^-1^ soil (S3 Table) whereas fungal 18S rRNA genes ranged from log_10_ 7.7 to 8.8 copies g^-1^ soil. Genes involved in soil-N cycling ranged from log_10_ 4.6 (AOB *amoA*) to 9.8 (*narG*) copies g^-1^ soil. AOB and AOA *amoA* genes were within the range of the other soil-N-cycling genes, except in two replicate plots (tree row of the Histosol and Anthrosol soils (grassland)) where these genes were below the detection limit. The ratio of fungal 18S-to-bacterial 16S rRNA gene abundances ranged from 0.01 to 0.1 across the two management systems of the studied soils and was higher in the tree rows than in the crop or grass rows of the agroforestry and the monoculture and open grassland system (p ≤ 0.01) (Fig 1).

### Genes involved in nitrification

**Fig 2:**
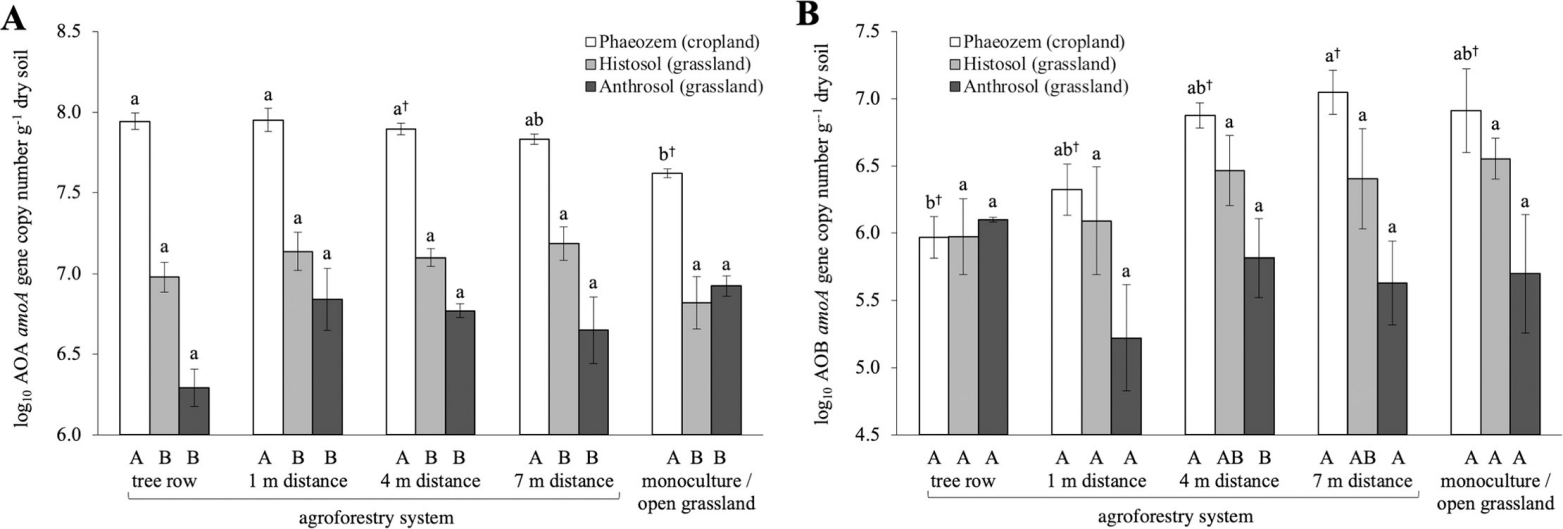
***amoA* gene abundances of (A) ammonia-oxidizing archaea (AOA) and (B) ammonia-oxidizing bacteria (AOB) in soils of paired temperate monoculture and agroforestry cropland, and paired temperate open grassland and agroforestry grassland.** Whiskers represent the SE (n *=* 4 for Phaeozem soil, n *=* 3 for Histosol and Anthrosol soils). Within the same soil type, means with different lowercase letters indicate significant differences among the tree row, 1 m, 4 m and 7 m within the grass or crop row of the agroforestry and the monoculture or open grassland system. Different uppercase letters indicate significant differences among soil types within the same sampling location of a management system (one-way ANOVA with Tukey’s HSD test or Kruskal-Wallis test with multiple comparison extension at p ≤ 0.05 and ^†^ p > 0.05 ≤ 0.07).

We quantified the gene abundance in two steps of nitrification (S2A Fig). Abundance of the AOA *amoA* gene in the Phaeozem soil (cropland) decreased with increasing distance from the tree row and was lowest in the monoculture system (p = 0.01) (Fig 2A). AOA *amoA* gene abundances were higher in the Phaeozem soil (cropland) compared to the grassland soils (Histosol and Anthrosol) in both management systems (p < 0.01). In contrast, AOB *amoA* gene abundance increased gradually with increasing distance from the tree row in the Phaeozem soil (cropland) (p = 0.06) (Fig 2B). In both grassland soils (Histosol and Anthrosol), no difference in AOA and AOB *amoA* gene abundances was observed between management systems. The ratio of AOA *amoA* to AOB *amoA* abundances was highly variable, ranging from 0.5 to 297. In 94% of the soil samples, AOA *amoA* genes outnumbered AOB *amoA* genes.

The abundance of nitrite oxidoreductase gene (*nxrB*) in the Phaeozem (cropland) and Histosol (grassland) soils did not differ among the tree row and the different distances within the crop or grass row of the agroforestry and the monoculture or open grassland systems (S3 Fig). In the tree row as well as at all distances within the crop or grass row of the agroforestry, *nxrB* gene abundance was higher in the Histosol soil (grassland) than in the Phaeozem soil (cropland) (p < 0.01–0.03) (S3 Fig). The ratio of ammonia monooxygenase (AOA+AOB *amoA*) to *nxrB* gene abundances in the Phaeozem soil (cropland) was on average more than 30 times larger than those in the Histosol and Anthrosol soils (grassland) (Fig 2 and S3 Fig).

### Genes involved in denitrification

**Fig 3:**
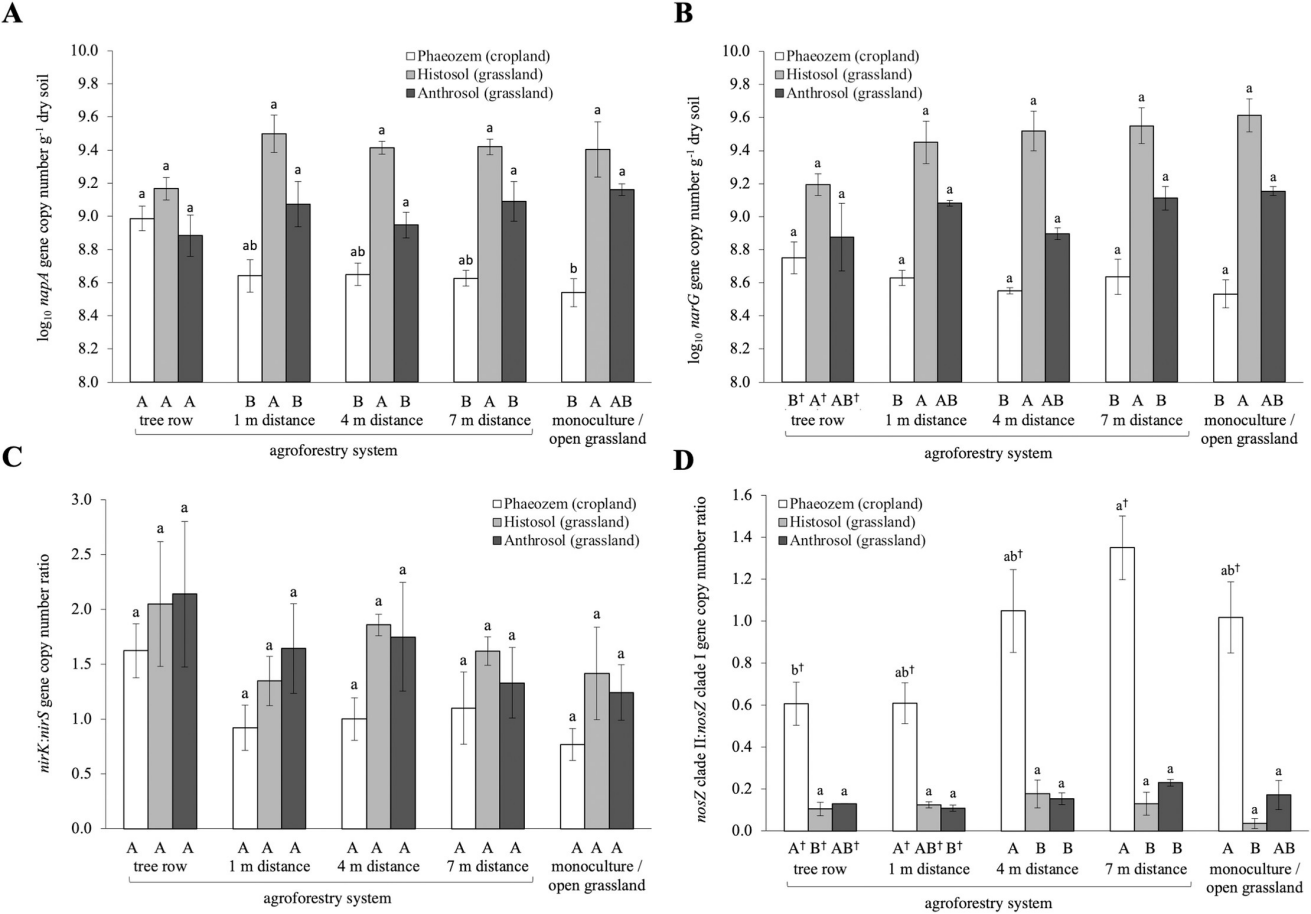
**Denitrification gene (A) *napA* and (B) *narG* abundances (C) and ratio of (C) *nirK-to-nirS* and (D) *nosZ* clade II-to-*nosZ* clade I abundances in soils of paired temperate monoculture and agroforestry cropland, and paired temperate open grassland and agroforestry grassland.** Whiskers represent the SE (n *=* 4 for Phaeozem soil, n *=* 3 for Histosol and Anthrosol soils). Within the same soil type, means with different lowercase letters indicate significant differences among the tree row, 1 m, 4 m and 7 m within the grass or crop row of the agroforestry and the monoculture or open grassland system. Different uppercase letters indicate significant differences among soil types within the same sampling location of a management system (one-way ANOVA with Tukey’s HSD test or Kruskal-Wallis test with multiple comparison extension at p ≤ 0.05 and ^†^ p > 0.05 ≤ 0.07).

We quantified the gene abundance that operate three specific steps in denitrification (S2B Fig). The abundance of the genes involved in nitrate reductase (*napA* and *narG*) did not differ among sampling locations within the agroforestry and the monoculture or open grassland system, except for the Phaeozem soil (cropland) where *napA* gene abundance in the tree row was higher than in the monoculture system (p = 0.05) (Fig 3A, Fig 3B). *napA* and *narG* gene abundances were higher in the Histosol soil (grassland) than the Phaeozem soil at all sampling locations (p < 0.01–0.06), except for *napA* in the tree row.

The abundance of nitrite reductase gene *nirK* in the agroforestry of Phaeozem soil (cropland) slightly decreased with increasing distance from the tree row and was lowest in the monoculture system (p = 0.07) (S4A Fig). The Histosol soil (grassland) showed higher *nirK* gene abundance than the Phaeozem soil at all sampling locations of the agroforestry system (p < 0.01–0.06). Gene abundances of *nirS* exhibited similar patterns as those of *nirK* gene among sampling locations and soil types (S4A Fig, S4B Fig). The ratio of *nirK*-to-*nirS* gene abundances did not differ among sampling locations within the agroforestry and the monoculture or open grassland system as well as among soil types (Fig 3C).

Lower abundance of nitrous oxide reductase gene *nosZ* clade I in the Phaeozem soil (cropland) than in the Histosol soil (grassland) was detected in all sampling locations of the agroforestry as well as in the monoculture or open grassland system (p < 0.01–0.04) (S4C Fig). In the tree row and at 4 m and 7 m within the grass row, *nosZ* clade I gene abundance was lower in the Anthrosol (grassland) than the Histosol soil (grassland) (p < 0.01) (S4C Fig). The abundance of *nosZ* clade II neither differed between management systems within each soil type nor among soil types, except between Phaeozem (cropland) and Anthrosol soils (grassland) for the tree rows (p = 0.07) (S4D Fig). The ratio of *nosZ* clade II-to-*nosZ* clade I gene abundances in the Phaeozem soil (cropland) was higher at 7 m within the crop row than in the tree row (p = 0.07) (Fig 3D). Additionally, the ratios of *nosZ* clade II-to-*nosZ* clade I gene abundances were also higher in the Phaeozem soil (cropland) than in the grassland soils (Histosol and Anthrosol) (p < 0.01–0.06) (Fig 3D).

### Relationships between gene abundance and soil properties

**Fig 4:**
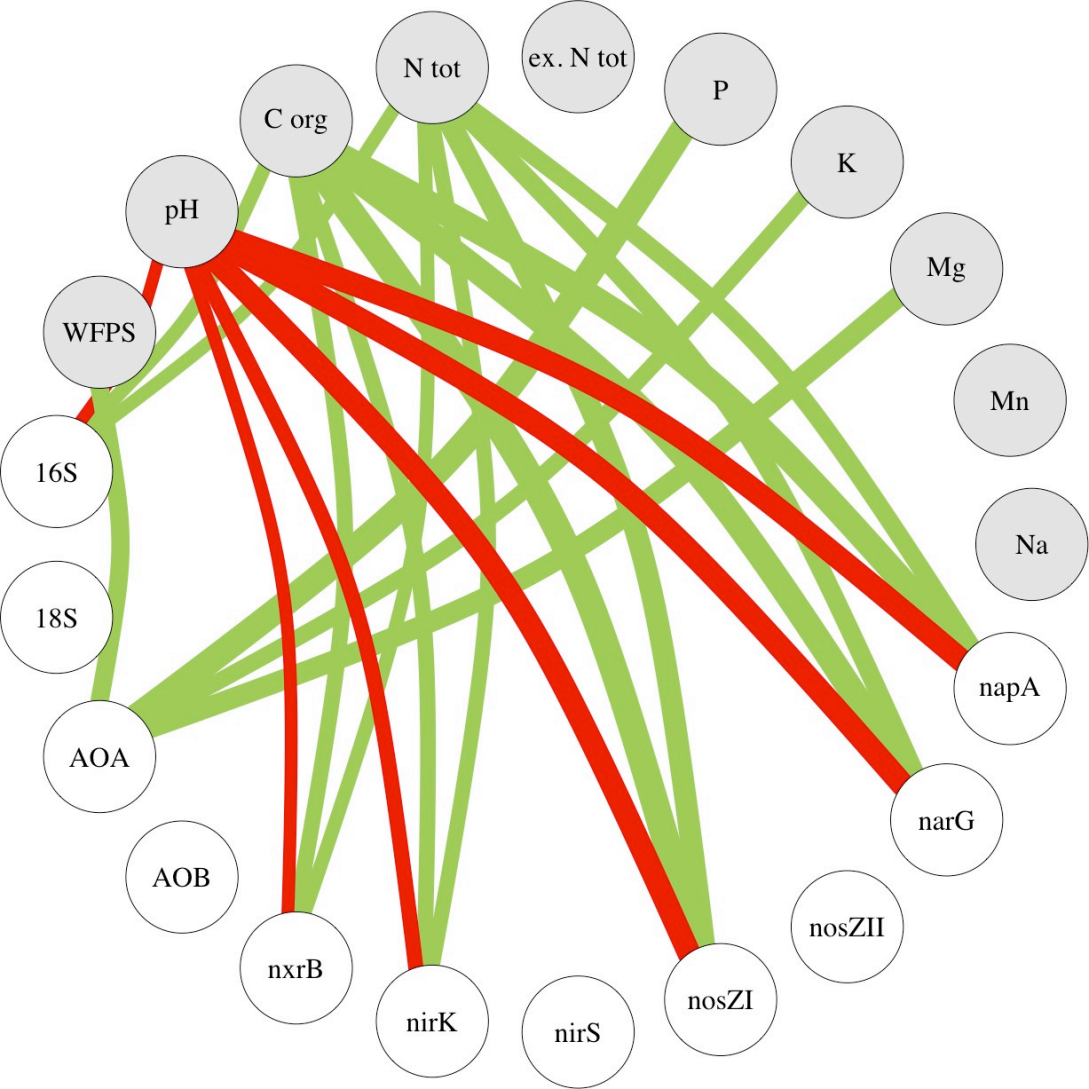
**Network analysis of the relationships between gene abundances (white nodes) and soil properties (gray nodes).** Lines are based on the thresholds of Spearman’s rank correlations, with r > |0.7| and p < 0.001 (S4 Table), across all replicate plots of paired temperate agroforestry and monoculture cropland or open grassland systems in all three soil types. Green lines represent positive and red lines represent negative correlations. The line’s width is proportional to the absolute magnitude of the correlation coefficient. 16S = bacterial 16S rRNA gene abundance, 18S = fungal 18S rRNA gene abundance, AOA = ammonia-oxidizing archaea *amoA* gene abundance, AOB = ammonia-oxidizing bacteria *amoA* gene abundance, nosZI = *nosZ* clade I gene abundance, NosZII = *nosZ* clade II gene abundance, P = plant-available P, ex. N tot = total extractable N, N tot = total N, C org = organic C, WFPS = water-filled pore space.

Bacterial 16S rRNA gene abundance was positively correlated to soil organic C and total N and negatively correlated to soil pH across all replicate plots (Fig 4 and S4 Table). AOA *amoA* gene abundance showed positive correlations to WFPS, plant-available P and exchangeable K and Mg. The correlations of gene abundances of *nxrB*, *napA*, *narG*, *nirK* and *nosZ* clade I with soil properties showed similar patterns: positive correlations with soil organic C and total N and negative correlation with soil pH. Compared to *nxrB* and *nirK*, stronger correlations were observed for *napA*, *narG* and *nosZ* clade I with these soil properties. Fungal 18S rRNA, AOB *amoA*, *nirS* and *nosZ* clade II showed only weaker correlations with the measured soil properties (S4 Table) and hence are not depicted in this network analysis. The PCA revealed similar pattern between gene abundance and soil properties (S5 Fig).

## Discussion

The increase in fungi-to-bacteria ratio in the tree row in all soil types (Fig 1) suggests that the tree component of agroforestry increased the relative fungal abundance. This finding concurred with the findings of Beuschel *et al*. [17] who reported larger fungal C-to-bacterial C ratios in the tree rows than the crop rows of poplar-based temperate agroforestry systems. As tree litter is more recalcitrant than crop or grass residues [54], increased tree litter likely accounts for the higher relative abundance of fungi in the agroforestry tree row. Moreover, tillage practiced in cropland reduces the growth of fungal hyphae and disrupts mycorrhizal networks [55–57], which may have contributed to the reduction of fungal abundance in the arable land (Fig 1). Other factors, such as changes in microclimate and theabsence of fertilization within the tree row, may also have contributed to the observed pattern. The ratio of fungal 18S-to-bacterial 16S rRNA gene abundances does not equal to a biomass ratio, as the copy number of rRNA genes is taxa-specific [58]. Such ratio, however, allows to detect shifts in the composition of microbial communities across soils [59].

The larger AOA than AOB *amoA* gene abundances (Fig 2) suggests that archaea were the predominant prokaryotes involved in ammonia oxidation at our study sites. Significant contribution of archaea to nitrification has been observed in previous studies (e.g. [60–63]). In contrast, recent studies demonstrated that although AOA *amoA* genes may numerically dominate over AOB *amoA*, enzymes encoded by AOB *amoA* genes control nitrification [64–67]. In the cropland on Phaeozem soil, the opposing trends of AOA and AOB *amoA* gene abundance with increasing distance from the tree row in the agroforestry and the large AOB in the monoculture system suggest a niche differentiation of AOA and AOB, e.g. due to their preference for an ammonium source [68,69]. We attributed this pattern of AOA and AOB niche differentiation to the influence of trees and the application of N fertilizer both in the agroforestry crop row and in the monoculture system. AOA abundance was shown to increase by mineralized ammonium derived from soil organic matter or low ammonium concentrations in soils [70,71], whereas AOB is favoured by ammonium applied as mineral fertilizer [71,72]. Thus, long-term absence of mineral N fertilization and cultivation as well as tree litter input within the tree row of the agroforestry system may have contributed to the opposite trends of AOA and AOB *amoA* gene abundance. The influence of other soil factors on the abundance of AOA and AOB populations under field conditions is still not extensively investigated, and it is likely that a combination of soil factors also influence AOA and AOB abundance as well as niche partitioning.

Apart from the ammonium source, the positive correlations of AOA and AOB *amoA* gene abundance with WFPS and plant-available P, K and Mg (Fig 4) across sites, although with weaker correlations for AOB (S4 Table), indicated that soil moisture and macronutrients may also influence the abundance of AOA and AOB *amoA* genes. Szukics *et al*. [73] claimed that the population adaptability of AOA is greater than of AOB, which would enable AOA to adapt rapidly to changing environmental conditions. We found that AOA *amoA* gene abundance was more responsive to soil moisture than AOB, based on the correlation coefficients (S4 Table). Plant-available P as well as K and Mg are rarely measured when quantifying genes involved in soil-N cycling. Previous studies showed that AOA are positively influenced by NaHCO_3_^-^ and Bray-extractable available P, whereas the response of AOB to available P was opposite [74–76]. Our findings that both AOA and AOB *amoA* gene abundances increased with increasing plant-available P, exchangeable K and Mg indicated a link between ammonium cycling and these rock-derived nutrients, which was not driven by the general bacterial and fungal genes since their abundance were not correlated with these nutrients (Fig 4, S4 Table).

There are only a limited number of studies to which we can relate the relative abundance of nitrite oxidoreductase *nxrB* gene to *amoA* genes. A possible explanation for the higher ratio of *amoA* (AOA+AOB) to *nxrB* gene abundances in the cropland soil (Phaeozem) than those in the grassland soils (Histosol and Anthrosol) may be the selectivity of our *nxrB* primer pair (NxrB-1F/R) for *Nitrobacter* strains [39,77]. The functional group of nitrite-oxidizing bacteria (NOB) is composed of six genera, namely *Nitrobacter*, *Nitrotoga*, *Nitrococcus*, *Nitrospina*, *Nitrospira* and *Nitrolancetus* [78]. Attard *et al*. [79] demonstrated that changes in nitrite oxidation are contributed by the shifts between *Nitrobacter*-like and *Nitrospira*-like NOB rather than within *Nitrobacter*-like populations. Thus, greater ratios between *amoA* and *nxrB* gene abundances in the Phaeozem soil (cropland) may originate from non-*Nitrobacter* dominated NOB populations, which cannot be assessed by our primer pair.

The increased *napA* gene abundances in the tree row of the Phaeozem soil (cropland) (Fig 3A) may indicate a more effective microbial removal of NO_3_^-^ in the tree row than the monoculture system. Root exudation of easily available C by trees is likely to facilitate microbial NO_3_^-^ removal through denitrification, dissimilatory NO_3_^-^ reduction and immobilization [80–82]. Apart from NO_3_^-^ uptake by tree roots, microbial NO_3_^-^ removal promoted by tree root exudates may additionally contribute to reduced NO_3_^-^ leaching in agroforestry systems. Overall, genes involved in denitrification were rather affected by soil type than management system (Fig 3A, Fig 3B, S4 Fig). The stark differences in *napA*, *narG*, *nirK* and *nosZ* clade I gene abundances among soil types across management systems (Fig 3A, Fig 3B, S4A Fig, S4C Fig), i.e. Histosol (grassland) and Anthrosol (grassland) > Phaeozem (cropland), were mainly attributed to higher soil organic C and total N as well as lower soil pH of the grassland than the cropland (Fig 4, S1 Table). Similar correlations were found for *nirS* gene, although with lower correlation coefficients (S4 Table), which agree with previous findings [83,84]. Studies reporting the relationships between the abundance of denitrifying genes and soil organic C and total N are contradictory, which may be attributed to different soil types and soil management practices [83,85–88]. Nevertheless, consistent with our findings, positive correlations between the abundances of *napA*, *narG*, *nirK*, *nirS* and *nosZ* clade I genes and soil organic C and total N have been observed in different ecosystems [85,86,88,89]. In addition, there are conflicting findings on the relationship between denitrification genes and soil pH, which relate to the range of pH covered in the studies. For example, increasing soil pH in acidic spruce forests exhibits a positive effect on *nirK* and a negative effect on *nirS* gene abundance [83], whereas the opposite was reported for grassland soils with a pH range from 6.4–7.1 [87]. Other studies even suggest that the abundance of both *nir* genes increases with increasing soil pH [84,90]. As the number of studies focusing on denitrifying genes and soil pH is limited and some studies cover rather narrow pH ranges, the effect of soil pH on the abundance of denitrification genes under field conditions is still not well understood.

Recent phylogenetic studies identified a previously overlooked clade of microorganisms harbouring *nosZ* [45,91]. This clade (*nosZ* clade II) has attracted great attention as its abundance and phylogenetic diversity has shown to be critical to the reduction of N_2_O to N_2_ in soils [92–94]. The *nosZ* clade II-type microorganisms cover a broad range of bacterial and archaeal phyla, whereas clade I-type microorganisms have been shown to consist exclusively of members of α-, β- and γ-proteobacteria [45]. The higher *nosZ* clade II-to-*nosZ* clade I ratio in the Phaeozem soil (cropland), signifying higher potential for N_2_O consumption, compared to the Histosol and Anthrosol soils (grassland) (Fig 3D) may suggest that this cropland soil may have a greater potential to consume atmospheric N_2_O to N_2_ relative to its potential for complete NO_3_^—^to-N_2_ reduction within the soil.

## Conclusion

The trees in our agroforestry systems increased the fungi-to-bacteria ratio and altered the abundance of AOA and AOB *amoA* genes (particularly in the cropland on a Phaeozem soil), suggesting a niche differentiation. These may be due to the long-term absence of fertilization and cultivation and tree litter input within the tree row of agroforestry. Abundances of genes encoding for nitrate (*napA* and *narG*) and nitrite reductase (*nirK* and *nirS*) as well as nitrous oxide reductase gene *nosZ* clade I were less affected by the management system than by soil type. Overall, our results show that temperate agroforestry can alter the abundance of soil bacteria and fungi and soil-N-cycling genes compared to monoculture and open grassland. It should be noted that this study relies on a single measurement period, which does not allow temporal extrapolation of our findings. Future studies should thus focus on the temporal dynamics of the genes involved in soil-N cycling and its controlling factors in order to gain deeper understanding of the services provided by trees in agroforestry systems.

## Supporting information

S1 Fig Map of the study sites in Germany. (A) study site locations and schematic illustration of the experimental setup. Borders of federal states are represented by black lines. Filled triangles represent the study sites. (B) and (C) are pictures taken within the grass or crop row of each agroforestry system.
(DOCX)

S2 Fig **Schematic diagram of genes encoding subunits of enzymes involved in (A) nitrification and (B) denitrification pathway.** Initial, intermediate and end product(s) of both pathways are connected by arrows labelled with subunits of genes commonly used for quantification. Genes on white arrows are the ones quantified in this study.
(DOCX)

S3 Fig *nxrB* gene abundances in soils of paired temperate monoculture and agroforestry cropland, and paired temperate open grassland and agroforestry grassland. Whiskers represent the SE (n *=* 4 for Phaeozem soil, n *=* 3 for Histosol and Anthrosol soils). Within the same soil type, means with different lowercase letters indicate significant differences among the tree row, 1 m, 4 m and 7 m within the grass or crop row of the agroforestry and the monoculture or open grassland system. Different uppercase letters indicate significant differences among soil types within the same sampling location of a management system (one-way ANOVA with Tukey’s HSD test or Kruskal-Wallis test with multiple comparison extension at p ≤ 0.05 and ^†^ p > 0.05 ≤ 0.06).
(DOCX)

S4 Fig **Denitrification gene (A) *nirK*, (B) *nirS*, (C) *nosZ* clade I, and (D) *nosZ* clade II abundances in soils of paired temperate monoculture and agroforestry cropland, and paired temperate open grassland and agroforestry grassland.** Whiskers represent the SE (n *=* 4 for Phaeozem soil, n *=* 3 for Histosol and Anthrosol soils). Within the same soil type, means with different lowercase letters indicate significant differences among the tree row, 1 m, 4 m and 7 m within the grass or crop row of the agroforestry and the monoculture or open grassland system. Different uppercase letters indicate significant differences among soil types within the same sampling location of a management system (one-way ANOVA with Tukey’s HSD test or Kruskal-Wallis test with multiple comparison extension at p ≤ 0.05 and ^†^ p > 0.05 ≤ 0.07).
(DOCX)

S5 Fig Two-dimensional principal component analysis biplot of gene abundances and soil properties across all replicate plots of paired temperate agroforestry and monoculture cropland or open grassland systems in all three soil types. Gene abundances and soil properties are represented by vectors, individual soil samples by triangles, circles and squares. 16S = bacterial 16S rRNA gene abundance, 18S = fungal 18S rRNA gene abundance, AOA = ammonia-oxidizing archaea amoA gene abundance, AOB = ammonia-oxidizing bacteria amoA gene abundance, nosZ I = nosZ clade I gene abundance, nosZ II = nosZ clade II gene abundance, P = plant-available P, ex. N tot = total extractable N, N tot = total N, C org = organic C, WFPS = water-filled pore space.
(DOCX)

S1 Table Soil properties of paired temperate monoculture and agroforestry cropland in a Phaeozem soil, and paired temperate open grassland and agroforestry grassland in a Histosol and Anthrosol soil. (DOCX)

S2 Table qPCR conditions and MgCl2 and primer concentration for each target gene. (DOCX)

S3 Table Maximum, minimum and mean of gene abundance across both management systems of cropland (Phaeozem soil) and grassland (Histosol & Anthrosol soils). (DOCX)

S4 Table Spearman’s rank correlation matrix between gene abundances and soil properties across all replicate plots in all three soil types. (DOCX)
