# Correction: Conversion of monoculture cropland and open grassland to agroforestry alters the abundance of soil bacteria, fungi and soil-N-cycling genes

**DOI:** 10.1371/journal.pone.0220713

**Published:** 2019-07-30

**Authors:** The PLOS ONE Staff

S1, S2, S3, S4 and S5 Figs are incorrect. Please view the correct Supporting Information files below. The publisher apologizes for the error.

## Supporting information

S1 Fig Map of the study sites in Germany. (A) study site locations and schematic illustration of the experimental setup. Borders of federal states are represented by black lines. Filled triangles represent the study sites. (B) and (C) are pictures taken within the grass or crop row of each agroforestry system.
(TIF)

S2 Fig **Schematic diagram of genes encoding subunits of enzymes involved in (A) nitrification and (B) denitrification pathway.** Initial, intermediate and end product(s) of both pathways are connected by arrows labelled with subunits of genes commonly used for quantification. Genes on white arrows are the ones quantified in this study.
(TIF)

S3 Fig *nxrB* gene abundances in soils of paired temperate monoculture and agroforestry cropland, and paired temperate open grassland and agroforestry grassland. Whiskers represent the SE (n *=* 4 for Phaeozem soil, n *=* 3 for Histosol and Anthrosol soils). Within the same soil type, means with different lowercase letters indicate significant differences among the tree row, 1 m, 4 m and 7 m within the grass or crop row of the agroforestry and the monoculture or open grassland system. Different uppercase letters indicate significant differences among soil types within the same sampling location of a management system (one-way ANOVA with Tukey’s HSD test or Kruskal-Wallis test with multiple comparison extension at p ≤ 0.05 and ^†^ p > 0.05 ≤ 0.06).
(TIF)

S4 Fig **Denitrification gene (A) *nirK*, (B) *nirS*, (C) *nosZ* clade I, and (D) *nosZ* clade II abundances in soils of paired temperate monoculture and agroforestry cropland, and paired temperate open grassland and agroforestry grassland.** Whiskers represent the SE (n = 4 for Phaeozem soil, n = 3 for Histosol and Anthrosol soils). Within the same soil type, means with different lowercase letters indicate significant differences among the tree row, 1 m, 4 m and 7 m within the grass or crop row of the agroforestry and the monoculture or open grassland system. Different uppercase letters indicate significant differences among soil types within the same sampling location of a management system (one-way ANOVA with Tukey’s HSD test or Kruskal-Wallis test with multiple comparison extension at p ≤ 0.05 and ^†^ p > 0.05 ≤ 0.07).
(TIF)

S5 Fig Two-dimensional principal component analysis biplot of gene abundances and soil properties across all replicate plots of paired temperate agroforestry and monoculture cropland or open grassland systems in all three soil types. Gene abundances and soil properties are represented by vectors, individual soil samples by triangles, circles and squares. 16S = bacterial 16S rRNA gene abundance, 18S = fungal 18S rRNA gene abundance, AOA = ammonia-oxidizing archaea amoA gene abundance, AOB = ammonia-oxidizing bacteria amoA gene abundance, nosZ I = nosZ clade I gene abundance, nosZ II = nosZ clade II gene abundance, P = plant-available P, ex. N tot = total extractable N, N tot = total N, C org = organic C, WFPS = water-filled pore space.
(TIF)
