# Supplementary material for: Conversion of monoculture cropland and open grassland to agroforestry alters the abundance of soil bacteria, fungi and soil-N-cycling genes
Source: PLoS One. 2019 Jun 27;14(6):e0218779. doi: 10.1371/journal.pone.0218779 (PMC6597161; doi:10.1371/journal.pone.0218779)
Supplement: S1 Fig — (A) study site locations and schematic illustration of the experimental setup. Borders of federal states are represented by black lines. Filled triangles represent the study sites. (B) and (C) are pictures taken within the grass or crop row of each agroforestry system. (DOCX) [file pone.0218779.s001.docx]

**S1 Fig. Map of the study sites in Germany**. (A) study site locations and schematic illustration of the experimental setup. Borders of federal states are represented by black lines. Filled triangles represent the study sites. (B) and (C) are pictures taken within the grass or crop row of each agroforestry system.
