# Supplementary material for: Conversion of monoculture cropland and open grassland to agroforestry alters the abundance of soil bacteria, fungi and soil-N-cycling genes
Source: PLoS One. 2019 Jun 27;14(6):e0218779. doi: 10.1371/journal.pone.0218779 (PMC6597161; doi:10.1371/journal.pone.0218779)
Supplement: S2 Fig — Schematic diagram of genes encoding subunits of enzymes involved in (A) nitrification and (B) denitrification pathway. Initial, intermediate and end product(s) of both pathways are connected by arrows labelled with subunits of genes commonly used for quantification. Genes on white arrows are the ones quantified in this study. (DOCX) [file pone.0218779.s002.docx]

**S2 Fig. Schematic diagram of genes encoding subunits of enzymes involved in (A) nitrification and (B) denitrification pathway.** Initial, intermediate and end product(s) of both pathways are connected by arrows labelled with subunits of genes commonly used for quantification. Genes on white arrows are the ones quantified in this study.
