# Supplementary material for: Conversion of monoculture cropland and open grassland to agroforestry alters the abundance of soil bacteria, fungi and soil-N-cycling genes
Source: PLoS One. 2019 Jun 27;14(6):e0218779. doi: 10.1371/journal.pone.0218779 (PMC6597161; doi:10.1371/journal.pone.0218779)
Supplement: S3 Fig — Whiskers represent the SE (n = 4 for Phaeozem soil, n = 3 for Histosol and Anthrosol soils). Within the same soil type, means with different lowercase letters indicate significant differences among the tree row, 1 m, 4 m and 7 m within the grass or crop row of the agroforestry and the monoculture or open grassland system. Different uppercase letters indicate significant differences among soil types within the same sampling location of a management system (one-way ANOVA with Tukey’s HSD test or Kruskal-Wallis test with multiple comparison extension at p ≤ 0.05 and † p > 0.05 ≤ 0.06). (DOCX) [file pone.0218779.s003.docx]

**S3 Fig. *nxrB* gene abundances in soils of paired temperate monoculture and agroforestry cropland, and paired temperate open grassland and agroforestry grassland.** Whiskers represent the SE (n *=* 4 for Phaeozem soil, n *=* 3 for Histosol and Anthrosol soils). Within the same soil type, means with different lowercase letters indicate significant differences among the tree row, 1 m, 4 m and 7 m within the grass or crop row of the agroforestry and the monoculture or open grassland system. Different uppercase letters indicate significant differences among soil types within the same sampling location of a management system (one-way ANOVA with Tukey’s HSD test or Kruskal-Wallis test with multiple comparison extension at p ≤ 0.05 and ^†^ p > 0.05 ≤ 0.06).
