# Supplementary material for: Conversion of monoculture cropland and open grassland to agroforestry alters the abundance of soil bacteria, fungi and soil-N-cycling genes
Source: PLoS One. 2019 Jun 27;14(6):e0218779. doi: 10.1371/journal.pone.0218779 (PMC6597161; doi:10.1371/journal.pone.0218779)
Supplement: S5 Fig — Gene abundances and soil properties are represented by vectors, individual soil samples by triangles, circles and squares. 16S = bacterial 16S rRNA gene abundance, 18S = fungal 18S rRNA gene abundance, AOA = ammonia-oxidizing archaea amoA gene abundance, AOB = ammonia-oxidizing bacteria amoA gene abundance, nosZ I = nosZ clade I gene abundance, nosZ II = nosZ clade II gene abundance, P = plant-available P, ex. N tot = total extractable N, N tot = total N, C org = organic C, WFPS = water-filled pore space. (DOCX) [file pone.0218779.s005.docx]

**S5 Fig. Two-dimensional principal component analysis biplot of gene abundances and soil properties across all replicate plots of paired temperate agroforestry and monoculture cropland or open grassland systems in all three soil types.** Gene abundances and soil properties are represented by vectors, individual soil samples by triangles, circles and squares. 16S = bacterial 16S rRNA gene abundance, 18S = fungal 18S rRNA gene abundance, AOA = ammonia-oxidizing archaea amoA gene abundance, AOB = ammonia-oxidizing bacteria amoA gene abundance, nosZ I = nosZ clade I gene abundance, nosZ II = nosZ clade II gene abundance, P = plant-available P, ex. N tot = total extractable N, N tot = total N, C org = organic C, WFPS = water-filled pore space.
