# Supplementary material for: Conversion of monoculture cropland and open grassland to agroforestry alters the abundance of soil bacteria, fungi and soil-N-cycling genes
Source: PLoS One. 2019 Jun 27;14(6):e0218779. doi: 10.1371/journal.pone.0218779 (PMC6597161; doi:10.1371/journal.pone.0218779)
Supplement: S1 Table — (DOCX) [file pone.0218779.s006.docx]

**S1 Table. Soil properties of paired temperate monoculture and agroforestry cropland in a Phaeozem soil, and paired temperate open grassland and agroforestry grassland in a Histosol and Anthrosol soil.**

| Soil properties^1^ | Management system | | | | |
| --- | --- | --- | --- | --- | --- |
|  | Agroforestry^2^ | | | | Monoculture /  open grassland |
|  | Tree row | 1 m distance | 4 m distance | 7 m distance |  |
|  | Phaeozem (cropland)^3^ | | | | |
| WFPS (%) | 70.4 ± 1.6 ab^†^ | 70.8 ± 3.5 ab^†^ | 66.5 ± 5.0 ab^†^ | 74.8 ± 3.0 a^†^ | 59.3 ± 3.9 b^†^ |
| Soil pH (1:4 in H_2_O) | 6.5 ± 0.0 b | 6.7 ± 0.1 b | 6.7 ± 0.0 b | 6.8 ± 0.0 b | 7.9 ± 0.1 a |
| Soil organic C (kg C m^-2^) | 1.0 ± 0.1 a | 0.9 ± 0.0 a | 0.8 ± 0.0 a | 0.9 ± 0.0 a | 0.8 ± 0.1 a |
| Total N (g N m^-2^) | 107.1 ± 4.9 a | 99.7 ± 1.5 a | 93.7 ± 5.6 a | 101.2 ± 4.2 a | 93.7 ± 11.5 a |
| Total extractable N (g N m^-2^) | 0.5 ± 0.1 a | 0.2 ± 0.0 a | 0.3 ± 0.0 a | 0.2 ± 0.0 a | 0.4 ± 0. a |
| Plant-available P (g P m^-2^) | 11.2 ± 1.0 a | 12.0 ± 0.8 a | 11.9 ± 0.4 a | 11.5 ± 0.2 a | 12.1 ± 1.1 a |
| Exchangeable K (g K m^-2^) | 20.3 ± 1.9 a | 19.0 ± 1.7 a | 18.8 ± 0.9 a | 18.8 ± 1.0 a | 22.6 ± 2.2 a |
| Exchangeable Mg (g Mg m^-2^) | 9.4 ± 0.5 ab^†^ | 9.0 ± 0.3 b^†^ | 9.8 ± 0.4 ab^†^ | 9.4 ± 0.7 ab^†^ | 20.26 ± 1.6 a^†^ |
| Exchangeable Mn (g Mn m^-2^) | 2.0 ± 0.2 a | 3.3 ± 2.2 ab | 0.9 ± 0.2 ab | 1.0 ± 0.2 ab | 0.1 ± 0.0 b |
| Exchangeable Na (g Na m^-2^) | 0.8 ± 0.2 a | 0.9 ± 0.1 a | 1.0 ± 0.1 a | 0.70 ± 0.1 a | 1.1 ± 0.2 a |
|  | Histosol (grassland)^3^ | | | | |
| WFPS (%) | 41.7 ± 16.3 a | 45.1 ± 7.7 a | 56.3 ± 12.3 a | 44.1 ± 20.5 a | 36.9 ± 12.0 a |
| Soil pH (1:4 in H_2_O) | 4.8 ± 0.2 a | 5.0 ± 0.2 a | 4.9 ± 0.1 a | 4.9 ± 0.1 a | 5.1 ± 0.1 a |
| Soil organic C (kg C m^-2^) | 4.2 ± 0.8 a | 4.7 ± 0.2 a | 6.4 ± 1.2 a | 6.9 ± 2.3 a | 5.2 ± 1.5 a |
| Total N (g N m^-2^) | 151.9 ± 16.9 a | 191.7 ± 3.9 a | 246.5 ± 29.9 a | 272.1 ± 90.7 a | 225.3 ± 58.3 a |
| Total extractable N (g N m^-2^) | 1.0 ± 0.4 a | 4.0 ± 3.4 a | 0.9 ± 0.1 a | 0.8 ± 0.3 a | 0.6 ± 0.2 a |
| Plant-available P (g P m^-2^) | 5.9 ± 0.9 a | 6.6 ± 0.4 a | 5.3 ± 0.6 a | 5.9 ± 1.8 a | 4.7 ± 0.7 a |
| Exchangeable K (g K m^-2^) | 1.8 ± 0.4 b | 2.4 ± 0.3 b | 4.0 ± 0.4 ab | 5.5 ± 1.2 a | 3.1 ± 0.5 ab |
| Exchangeable Mg (g Mg m^-2^) | 2.7 ± 0.2 b | 4.4 ± 0.7 b | 4.3 ± 0.5 b | 6.6 ± 0.6 a | 3.5 ± 0.1 b |
| Exchangeable Mn (g Mn m^-2^) | 1.5 ± 0.3 a | 3.5 ± 0.9 a | 3.3 ± 0.3 a | 4.0 ± 1.0 a | 2.7 ± 0.3 a |
| Exchangeable Na (g Na m^-2^) | 0.3 ± 0.2 a | 0.5 ± 0.3 a | 0.3 ± 0.1 a | 0.7 ± 0.1 a | 0.4 ± 0.1 a |
|  | Anthrosol (grassland)^3^ | | | | |
| WFPS (%) | 11.8 ± 1.9 a | 14.4 ± 1.2 a | 16.2 ± 2.0 a | 17.9 ± 5.4 a | 12.1 ± 1.6 a |
| Soil pH (1:4 in H_2_O) | 5.8 ± 0.0 b^†^ | 5.9 ± 0.1 ab^†^ | 5.9 ± 0.1 ab^†^ | 5.9 ± 0.0 ab^†^ | 6.0 ± 0.0 a^†^ |
| Soil organic C (kg C m^-2^) | 2.3 ± 0.1 a | 3.0 ± 0.2 a | 3.1 ± 0.7 a | 3.0 ± 0.9 a | 2.3 ± 0.2 a |
| Total N (g N m^-2^) | 129.5 ± 7.0 a | 175.9 ± 5.1 a | 195.3 ± 33.5 a | 183.7 ± 54.4 a | 148.9 ± 10.8 a |
| Total extractable N (g N m^-2^) | 0.3 ± 0.1 a | 0.3 ± 0.1 a | 0.3 ± 0.0 a | 0.4 ± 0.1 a | 0.2 ± 0.0 a |
| Plant-available P (g P m^-2^) | 2.3 ± 0.1 a | 2.7 ± 0.2 a | 2.6 ± 0.5 a | 2.7 ± 0.8 a | 2.4 ± 0.2 a |
| Exchangeable K (g K m^-2^) | 1.7 ± 0.2 a | 3.2 ± 0.5 a | 2.6 ± 0.3 a | 2.6 ± 0.2 a | 2.2 ± 0.3 a |
| Exchangeable Mg (g Mg m^-2^) | 2.0 ± 0.2 a | 3.2 ± 0.3 a | 3.2 ± 0.5 a | 3.0 ± 0.3 a | 2.5 ± 0.6 a |
| Exchangeable Mn (g Mn m^-2^) | 0.8 ± 0.1 a | 1.0 ± 0.1 a | 1.0 ± 0.1 a | 0.9 ± 0.1 a | 0.7 ± 0.1 a |
| Exchangeable Na (g Na m^-2^) | 0.4 ± 0.1 a | 0.5 ± 0.1 a | 0.6 ± 0.1 a | 0.5 ± 0.1 a | 0.3 ± 0.1 a |

Soil pH, soil organic C, total N, exchangeable K, Mg, Mn, Na were measured in 2016, water-filled pore space (WFPS), total extractable N and plant-available P were measured in 2017 on the same day that soil samples for DNA extraction were taken. Values are for the top 0.05-m soil depth. ^1^Soil data are provided by Göbel *et al*. (unpublished data) and Schmidt *et al*. (unpublished data). ^2^ 1 m, 4 m and 7 m distance are distances from the tree row to the crop or grass row within the cropland or grassland agroforestry. ^3^ Means ± SE (n = 4 for Phaeozem, n = 3 for Histosol & Anthrosol) followed by a different lowercase letter indicate significant differences among sampling locations within the agroforestry and the monoculture or open grassland system (one-way ANOVA with Tukey’s HSD test or Kruskal-Wallis test with multiple comparison extension at p ≤ 0.05 and ^†^ p > 0.05 ≤ 0.08).
