# Supplementary material for: Conversion of monoculture cropland and open grassland to agroforestry alters the abundance of soil bacteria, fungi and soil-N-cycling genes
Source: PLoS One. 2019 Jun 27;14(6):e0218779. doi: 10.1371/journal.pone.0218779 (PMC6597161; doi:10.1371/journal.pone.0218779)
Supplement: S2 Table — (DOCX) [file pone.0218779.s007.docx]

**S2 Table. qPCR conditions and MgCl_2_ and primer concentration for each target gene.**

| Target gene | Initial denaturation | 35 cycles | | | MgCl_2_ concentration (mM) | Primer concentration (µM) |
| --- | --- | --- | --- | --- | --- | --- |
|  |  | Denaturation | Annealing | Extension |  |  |
| Bacterial  16S rRNA | 95°C, 120s | 94°C, 20s | 60°C, 30s | 68°C, 30s | 2.5 | 0.3 |
| Fungal  18S rRNA | 95°C, 120s | 94°C, 20s | 55°C, 30s | 68°C, 30s | 2.5 | 0.3 |
| AOA *amoA* | 95°C, 120s | 94°C, 20s | 61°C, 30s | 68°C, 45s | 3.0 | 0.3 |
| AOB *amoA* | 95°C, 120s | 94°C, 20s | 60°C, 30s | 68°C, 35s | 2.0 | 0.3 |
| *nxrB* | 95°C, 120s | 94°C, 20s | 63°C, 30s | 68°C, 35s | 2.5 | 0.3 |
| *napA* | 95°C, 120s | 94°C, 20s | 61°C, 30s | 68°C, 15s | 2.0 | 0.5 |
| *narG* | 95°C, 120s | 94°C, 20s | 58°C, 30s | 68°C, 15s | 2.5 | 0.5 |
| *nirK* | 95°C, 120s | 94°C, 20s | 58°C, 30s | 68°C, 30s | 2.5 | 0.5 |
| *nirS* | 95°C, 120s | 94°C, 20s | 53°C, 30s | 68°C, 30s | 2.0 | 0.5 |
| *nosZ* clade I | 95°C, 120s | 94°C, 20s | 60°C, 30s | 68°C, 30s | 1.5 | 0.5 |
| *nosZ* clade II | 95°C, 120s | 94°C, 20s | 58°C, 30s | 68°C, 45s | 2.0 | 1.0 |
