# Supplementary material for: Conversion of monoculture cropland and open grassland to agroforestry alters the abundance of soil bacteria, fungi and soil-N-cycling genes
Source: PLoS One. 2019 Jun 27;14(6):e0218779. doi: 10.1371/journal.pone.0218779 (PMC6597161; doi:10.1371/journal.pone.0218779)
Supplement: S3 Table — (DOCX) [file pone.0218779.s008.docx]

**S3 Table. Maximum, minimum and mean of gene abundance across both management systems of cropland (Phaeozem soil) and grassland (Histosol & Anthrosol soils).**

| Target gene | log_10_ gene copy number g^-1^ dry soil | | |
| --- | --- | --- | --- |
|  | Maximum | Minimum | Mean |
| Bacterial 16S rRNA | 10.3 | 9.3 | 9.9 |
| Fungal 18S rRNA | 8.8 | 7.7 | 8.3 |
| AOA *amoA* ^a^ | 8.1 | 6.1 | 7.3 |
| AOB *amoA* ^a^ | 7.5 | 4.6 | 6.3 |
| *nxrB* | 7.5 | 5.9 | 6.8 |
| *napA* | 9.7 | 8.4 | 9.0 |
| *narG* | 9.8 | 8.4 | 9.0 |
| *nirK* | 8.9 | 7.7 | 8.4 |
| *nirS* | 8.7 | 7.9 | 8.3 |
| *nosZ* clade I | 8.9 | 7.6 | 8.2 |
| *nosZ* clade II | 8.1 | 6.3 | 7.6 |

^a^ calculated without the one replicate that was below the detection limit.
