# Supplementary material for: Conversion of monoculture cropland and open grassland to agroforestry alters the abundance of soil bacteria, fungi and soil-N-cycling genes
Source: PLoS One. 2019 Jun 27;14(6):e0218779. doi: 10.1371/journal.pone.0218779 (PMC6597161; doi:10.1371/journal.pone.0218779)
Supplement: S4 Table — (DOCX) [file pone.0218779.s009.docx]

**S4 Table.** **Spearman’s rank correlation matrix between gene abundances and soil properties across all replicate plots in all three soil types.**

| Soil properties | Gene abundance (gene copies g^-1^ dry soil) | | | | | | | | | | |
| --- | --- | --- | --- | --- | --- | --- | --- | --- | --- | --- | --- |
|  | 16S rRNA^a^ | 18S rRNA^b^ | AOA^c^ *amoA* | AOB^d^ *amoA* | *nxrB* | *napA* | *narG* | *nirK* | *nirS* | *nosZ* clade I | *nosZ* clade II |
| WFPS^e^ (%) | -0.33^*^ | 0.05 | 0.79^***^ | 0.54^***^ | -0.16 | -0.21 | -0.29^*^ | -0.37^**^ | -0.30^*^ | -0.32^*^ | 0.43^**^ |
| Soil pH (1:4 H_2_O) | -0.74^***^ | -0.44^**^ | 0.60^***^ | 0.39^**^ | -0.74^***^ | -0.83^***^ | -0.83^***^ | -0.77^***^ | -0.51^***^ | -0.83^***^ | 0.37^**^ |
| Soil organic C  (g C m^-2^) | 0.76^***^ | 0.39^**^ | -0.58^***^ | -0.34 | 0.77^***^ | 0.84^***^ | 0.84^***^ | 0.77^***^ | 0.63^***^ | 0.85^***^ | -0.28 |
| Total N (g N m^-2^) | 0.75^***^ | 0.28 | -0.61^***^ | -0.39^**^ | 0.75^***^ | 0.79^***^ | 0.80^***^ | 0.77^***^ | 0.67^***^ | 0.82^***^ | -0.28^*^ |
| Total extractable total N (g N m^-2^) | 0.32^*^ | 0.29^*^ | -0.12 | 0.00 | 0.42^**^ | 0.57^***^ | 0.46^***^ | 0.27 | 0.26 | 0.40^**^ | -0.12 |
| Plant-available P  (g P m^-2^) | -0.51^***^ | -0.08 | 0.84^***^ | 0.49^***^ | -0.41^**^ | -0.46^***^ | -0.52^***^ | -0.62^***^ | -0.40^**^ | -0.54^***^ | 0.46^***^ |
| Exchangeable K  (g K m^-2^) | -0.57^***^ | -0.18 | 0.78^***^ | 0.48^**^ | -0.45^**^ | -0.52^***^ | -0.55^***^ | -0.60^***^ | -0.40^**^ | -0.59^***^ | 0.47^**^ |
| Exchangeable Mg  (g Mg m^-2^) | -0.50^***^ | -0.15 | 0.81^***^ | 0.48^**^ | -0.42^***^ | -0.49^***^ | -0.53^***^ | -0.59^***^ | -0.36^**^ | -0.57^***^ | 0.55^***^ |
| Exchangeable Mn  (g Mn m^-2^) | 0.87^**^ | 0.38^**^ | -0.09 | -0.06 | 0.48^**^ | 0.57^***^ | 0.58^***^ | 0.38^**^ | 0.35^**^ | 0.51^***^ | -0.03 |
| Exchangeable Na  (g Na m^-2^) | -0.39^**^ | -0.21 | 0.54^***^ | 0.18 | -0.33^*^ | -0.42^**^ | -0.44^**^ | -0.43^**^ | -0.17 | -0.45^**^ | 0.28^*^ |

Soil pH, soil organic C, total N, exchangeable K, Mg, Mn, Na were measured in 2016; water-filled pore space (WFPS), extractable total N and plant-available P were measured in 2017 on the same day that soil samples for DNA extraction were taken. ^a^ bacterial 16S rRNA gene, ^b^ fungal 18S rRNA gene, ^c^ ammonia-oxidizing archaea, ^d^ ammonia-oxidizing bacteria, ^e^ water-filled pore space, ^*^ p ≤ 0.05, ^**^ p ≤ 0.01, ^***^ p < 0.001.
